# SH3 domain regulation of RhoGAP activity: Crosstalk between p120RasGAP and DLC1 RhoGAP

**DOI:** 10.1038/s41467-022-32541-4

**Published:** 2022-08-15

**Authors:** Jocelyn E. Chau, Kimberly J. Vish, Titus J. Boggon, Amy L. Stiegler

**Affiliations:** 1 Department of Molecular Biophysics and Biochemistry, Yale University, New Haven, CT USA; 2 Department of Pharmacology, Yale University, New Haven, CT USA

**Keywords:** X-ray crystallography, GTP-binding protein regulators, RHO signalling

## Abstract

RhoGAP proteins are key regulators of Rho family GTPases and influence a variety of cellular processes, including cell migration, adhesion, and cytokinesis. These GTPase activating proteins (GAPs) downregulate Rho signaling by binding and enhancing the intrinsic GTPase activity of Rho proteins. Deleted in liver cancer 1 (DLC1) is a tumor suppressor and ubiquitously expressed RhoGAP protein; its activity is regulated in part by binding p120RasGAP, a GAP protein for the Ras GTPases. In this study, we report the co-crystal structure of the p120RasGAP SH3 domain bound directly to DLC1 RhoGAP, at a site partially overlapping the RhoA binding site and impinging on the catalytic arginine finger. We demonstrate biochemically that mutation of this interface relieves inhibition of RhoGAP activity by the SH3 domain. These results reveal the mechanism for inhibition of DLC1 RhoGAP activity by p120RasGAP and demonstrate the molecular basis for direct SH3 domain modulation of GAP activity.

## Abstract (web summary)

Inhibition of DLC1 RhoGAP by p120RasGAP allows for crosstalk between the Rho and Ras pathways. Here, Chau et al. present the co-crystal structure of DLC1 RhoGAP domain and p120RasGAP SH3 domain and probe the interaction with biochemical studies.

## Introduction

The Ras superfamily of small GTPases are molecular switches that cycle between GTP- and GDP-bound states in response to stimuli. These nucleotide-binding states reflect the “on” and “off” signaling conformations of these proteins, respectively. The GTPase cycle is modulated by the guanine nucleotide exchange factors (GEFs), which facilitate the exchange of GDP for GTP to activate GTPase signaling, and the GTPase activating proteins (GAPs), which enhance the intrinsic GTP hydrolysis activity of the GTPases and downregulate GTPase signaling^1^. The Rho (Ras homolog) subfamily of small GTPases, including the prominent members RhoA, Cdc42, and Rac1, function in actin cytoskeleton dynamics to affect such cellular processes as cell migration, adhesion, and morphology. Additionally, they act in cell cycle progression, vesicular trafficking, and endocytosis among other functions^2^.

Activation of Rho GTPase signaling is facilitated by Rho-family specific RhoGEFs, including the Dbl-homology (DH) domain-containing proteins such as SOS and BCR, while downregulation of Rho GTPase signaling is provided by specific RhoGAP proteins. In addition, Rho GDP dissociation inhibitors (GDIs) also downregulate Rho activity by preventing exchange of nucleotide and membrane localization of the GTPases (reviewed in^3,4^). RhoGAPs enhance the intrinsically inefficient catalytic activity by directly binding the GTP-bound GTPase and contributing a conserved arginine residue, the so-called catalytic “arginine finger”, to stabilize the transition state of the GTPase enzyme active site and catalyze the reaction^5,6^. The human genome encodes nearly 70 RhoGAPs, including p50RhoGAP, the p190RhoGAP proteins, and the Graf, ARAP, chimaerin, and DLC proteins^4,7^. Many of these RhoGAPs are multidomain proteins, and these domains are implicated in regulation of GAP activity as well as scaffolding functions for crosstalk with other signaling pathways^8^. Though some RhoGAPs are suggested to be regulated by mechanisms including localization and post-translational modification, much remains to be discovered at the molecular level regarding how their RhoGAP activities are controlled^4^.

**Fig. 1:**
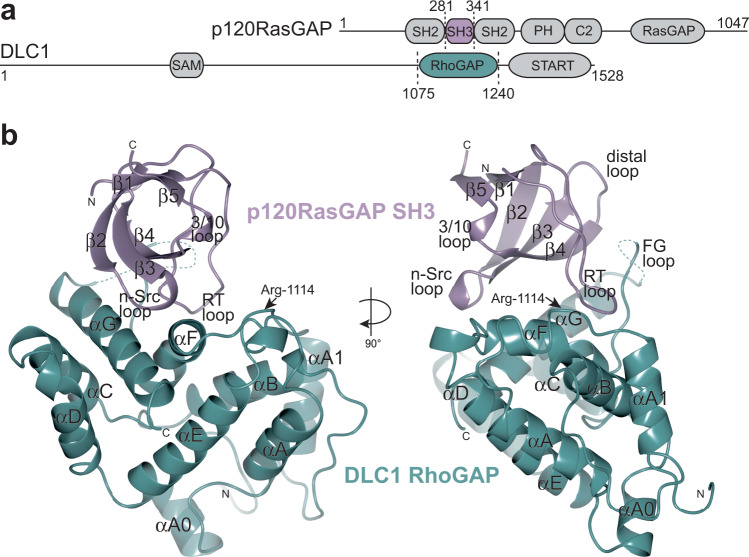
Complex of the SH3 domain of p120RasGAP and the RhoGAP domain of DLC1. **a** Domain architecture of p120RasGAP (Uniprot ID: P20936. Domain IDs: SH2: Src Homology 2, SH3: Src Homology 3, PH: Pleckstrin Homology, C2: Protein Kinase C Conserved domain 2; RasGAP: Ras GTPase Activating Protein) and DLC1 (Uniprot ID: Q96QB1, SAM: Sterile Alpha Motif, RhoGAP: Rho GTPase Activating Domain, START: steroidogenic acute regulatory-related lipid transfer). Residue numbers are indicated. Domains from each protein co-crystallized are highlighted in color. **b** Ribbon diagram of the co-crystal of p120RasGAP SH3 domain (purple) in complex with DLC1 RhoGAP domain (teal). Labeled are the secondary structure elements, loops, amino- and carboxy-termini, and the position of Arg-1114 in DLC1 indicated with an arrow.

The *DLC1* gene (deleted in liver cancer 1, *STARD12*, *ARHGAP7*) encodes a ubiquitously expressed Rho GTPase-activating protein that is a tumor suppressor and can regulate RhoA activity both in vitro and in vivo^9,10^. *DLC1* is the prototypical member of a family that in mammals includes the related genes *DLC2* (*STARD13*) and *DLC3* (*STARD8*), but in worm and fly is conserved as a single DLC gene^11–14^. It was first identified as a gene deleted in multiple primary hepatocellular carcinoma (HCC) tumors and liver cancer cell lines^15^ and upon cloning was observed to be homologous to the rat p122RhoGAP^16^. Deletion of DLC1 is embryonic lethal, and thus its critical role in cytoskeletal organization was established^17^. DLC1 has been shown to act on Rho family GTPases RhoA, RhoB, and RhoC, and to a lesser extent on Cdc42, Rac1 and TC10^7,9^. In addition to the RhoGAP domain located in their central region, each of the DLC proteins contain a sterile alpha motif (SAM) domain in their N-terminus, and a steroidogenic acute regulatory-related lipid transfer (START) domain near their C-terminus (Fig. 1a^11,15^). A serine-rich linker region (LR) between the SAM and RhoGAP domains is also present and includes a focal adhesion targeting (FAT) region. Localization of DLC proteins and regulation of the RhoGAP domain are thought to be mediated by the accessory domains^9,18^, and by partner proteins, including tensin proteins, focal adhesion kinase and talin^19–24^. However, the molecular mechanisms of regulation and targeting remain poorly understood.

One major binder and regulator of DLC1 is the RasGAP protein, p120RasGAP (*RASA1*; RasGAP, p120GAP). This was the first RasGAP identified and is a key regulator of Ras signaling^25–28^. p120RasGAP is a multidomain protein containing two Src homology-2 (SH2), one Src-homology-3 domain (SH3), a Pleckstrin homology (PH) domain, presumptive calcium-binding C2 domain, and a RasGAP domain (Fig. 1a). It is recruited to phosphotyrosine containing proteins by its SH2 domains, and the PH and C2 domains are thought to mediate membrane localization^29^; however, the role of the SH3 domain is non-canonical, as it has not been demonstrated to bind to poly-proline (PxxP) motifs^30,31^. Instead, the SH3 domain was first observed by yeast two hybrid to bind the DLC1 RhoGAP domain and suppress its RhoGAP activity^32^, but the molecular mechanism was not determined^33^.

The roles and divergence of Src homology domains away from their canonical features continue to be identified^34–36^, including the recent discovery of a unique mode of phosphotyrosine binding by one of the SH2 domains of p120RasGAP^37^. We, therefore, hypothesized that the interaction of the p120RasGAP SH3 domain with the DLC1 RhoGAP domain may represent an atypical mode of SH3 binding partner recognition. Furthermore, direct regulation of a GTPase activating protein by an SH3 domain, one of the most frequent domain ‘modules’ in the human genome with over 330 sequences^38^, may represent a previously under-appreciated mode of control for GTPase signaling cascades. We, therefore, determined the co-crystal structure of DLC1 RhoGAP domain in complex with p120RasGAP SH3 domain. This structure of a RhoGAP-SH3 interaction reveals that the RhoGAP domain cradles the SH3 domain in a manner competitive with its binding to RhoA. Nonetheless, the binding sites encompass different regions of the RhoGAP domain, allowing site directed mutagenesis in the SH3 binding site to selectively disrupt DLC1 suppression by p120RasGAP without impinging on RhoGAP activity. Overall, this study reveals the molecular basis of a noncanonical role for SH3 domains in regulation and signaling of small GTPase signaling cascades.

## Results

### Co-crystal structure of p120RasGAP SH3 domain with DLC1 RhoGAP domain

To reveal the molecular basis of SH3 binding and suppression of GAP activity, we sought a co-crystal structure of p120RasGAP SH3 domain and DLC1 RhoGAP domain. We recombinantly expressed the isolated DLC1 RhoGAP and p120RasGAP SH3 domains, and copurified the complex. We determined the co-crystal structure to 3.2 Å resolution (Fig. 1 and Table 1), and observe clear electron density throughout, including the key residues that mediate the protein-protein interaction (Supplementary Fig. 1). There are 4 copies of the DLC1-p120RasGAP complex found in the asymmetric unit, and they are experimentally identical (RMSD between 0.3 and 0.6 Å over 251 Cα atoms) (Supplementary Fig. 2a). This structure thus provides an experimentally determined molecular-level structure describing an interaction between a RhoGAP and SH3 domain.

**Table 1:** Data collection and refinement statistics

Data collection	DLC1 RhoGAP + p120RasGAP SH3
PDB accession code	7TPB
Wavelength (Å)	0.97918
Resolution range (Å)	50–3.20 (3.31–3.20)
Space group	*H* 3
Cell dimensions *a*, *b*, *c* (Å)	143.7, 143.7, 152.8
*α*, *β*, *γ* (°)	90, 90, 120
Unique reflections	19420
Multiplicity	9.1 (7.8)
Completeness (%)	100 (100)
Mean *I*/σ*I*	9.9 (1.6)
Wilson B factor (Å^2^)	80.4
*R*_pim_ (%)	9.0 (65.7)
CC½	99.3 (33.2)
CC^∗^	99.8 (70.6)
**Refinement**	
Resolution range (Å)	48.25–3.20 (3.37–3.20)
Reflections used in refinement	18492 (2658)
Reflections used for *R*_free_	920 (122)
% Reflections used for *R*_free_	4.7 (4.6)
*R*_work_ (%)	21.2 (30.1)
*R*_free_ (%)	26.0 (33.2)
**No. of non-hydrogen atoms**	
Protein	8096
RMSD	
Bond lengths (Å)	0.002
Bond angles (°)	0.474
**Ramachandran plot**	
Favored, allowed, outliers (%)	99.2, 0.8, 0
Rotamer outliers (%)	0
MolProbity clashscore	5.7 (100th percentile)
**Average** ***B*** **factor (Å**^**2**^**)**	
Overall	85.5
Copies A, C, E, G (SH3)	84.6, 92.8, 101.5, 105.7
Copies B, D, F, H (RhoGAP)	73.5, 80.6, 83.2, 91.2

Statistics for the highest resolution shell are shown in parentheses. *RMSD* root-mean-square deviation.

The DLC1 RhoGAP domain (residues 1075–1240) adopts a conserved overall structure that is highly similar to the GAP domain of other RhoGAP proteins, including the previously determined structure of the isolated DLC1 RhoGAP domain (PDB ID: 3KUQ^39^). Its closest structural neighbor is the GAP domain of p50RhoGAP (PDB ID: 1TX4^40^, RMSD 2.0 over 188 Cα atoms, 27% identity, Dali server, Supplementary Fig. 2b). The DLC1 RhoGAP domain consists of nine helices, termed αA0, αA, αA1, αB, αC, αD, αE, αF, and αG according to the nomenclature of Barrett et al.^41^. At the core of the domain is a four-helix bundle of αA-αB and αE-αF. αA-αB is flanked by αA1 and capped by αA0. αC-αD form a hairpin that protrudes from the four-helix bundle and, together with αE-αF, forms a V-shaped groove for αG. The long FG loop (residues 1234–1259), which is of variable length, sequence and structure in the RhoGAP family^7^, is partially disordered in this structure (Fig. 1b) and is located adjacent to the solvent channel in all four copies. In GAP proteins, the critical catalytic residue is a highly conserved arginine in the αA-αA1 loop (or the “finger loop”), termed the arginine finger^5,6^. In DLC1 RhoGAP this residue is Arg-1114. Comparison with the unpublished structure of apo DLC1 RhoGAP (PDB ID: 3KUQ^39^) shows only minor conformational changes (RMSD value of 0.7 Å over 186 Cα atoms), potentially due to crystal packing and distal from the SH3 binding site (residues 1130–1134 in αA1-αB located approximately 20–30 Å from the SH3 binding site, Supplementary Fig. 2c).

The p120RasGAP SH3 domain (residues 281–341) adopts a beta sandwich fold comprised of five strands (β1-β5) that form a β-barrel. The strands are connected by loops termed the RT loop (β1-β2), n-Src loop (β2-β3), distal loop (β3-β4), and a 3/10 helix (β4-β5) (based on Src nomenclature^42^). Several previous structure determinations of the apo form of p120RasGAP SH3 domain have been reported: PDB IDs: 2J05 and 2J06^43^, 4FSS (crystal structure, unpublished), 2GQI (NMR, unpublished), and 2M51 (NMR, unpublished). The structure here superposes well with these previous structures with RMSD values ranging from 0.4 to 0.8 Å over 58 equivalent Cα positions (Supplementary Fig. 2d). We observe no material conformational change in either the SH3 domain or RhoGAP domain upon complex formation when compared to the previous apo structures. Therefore, the binding mode can be defined as ‘lock-and-key’ type rather than as an ‘induced fit’ interaction.

**Fig. 2:**
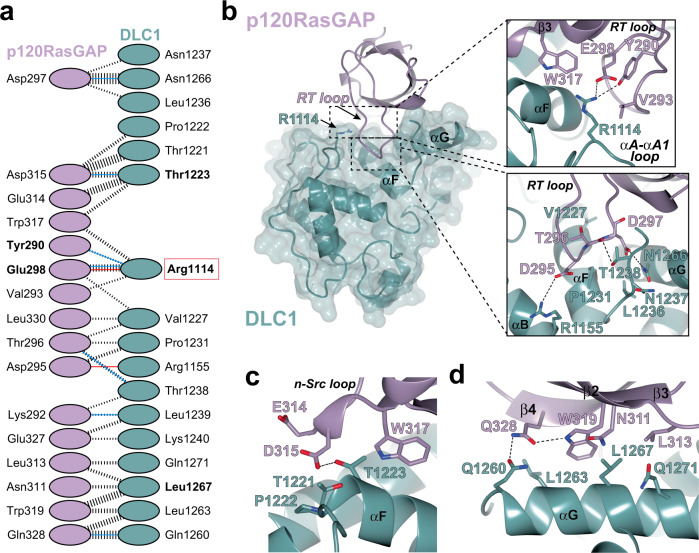
Details of interface between DLC1 and p120RasGAP. **a** Residue interactions adapted from PDBSum^86^ and PISA server^44^. Blue line indicates hydrogen bond, red line indicates salt bridge. The width of the black dashed lines is proportional to the number of non-bonded atomic contacts as calculated in PDBSum^86^. SH3 domain in purple and RhoGAP domain in teal. Residues mutated in this study are labeled in bold. **b** RT loop binding site. Insets show details of the interactions. Details of the interaction showing the SH3 domain contacts made by the n-Src loop (**c**) and β2-β3-β4 strands with αG (**d**).

The complex between the SH3 domain of p120RasGAP and the RhoGAP domain of DLC1 represents a noncanonical mechanism for SH3 domain recognition of binding partners, and for RhoGAP domains to be inhibited. The p120RasGAP SH3 domain latches onto the DLC1 RhoGAP domain using its RT and n-Src loops and additional contacts are made between these latches by residues in the β2, β3 and β4 strands (Fig. 1b). On the RhoGAP domain, the bulk of the interface comprises residues on the solvent-accessible faces of αF and αG, with several more contributions from the αA-αA1 loop, αB, and the FG loop (Fig. 1b). Mapping the interaction regions on the surface of the two proteins reveals a single continuous binding site that buries a total surface area of 1550 Å^2^ comprising approximately 8% of the DLC1RhoGAP surface and 20% of the SH3 surface, with a shape complementarity score of 1.00 (Pisa server^44^, Fig. 2a). The interface is largely hydrophobic, yet also involves several negatively charged residues from SH3 and positively charged surface on RhoGAP.

### Details of binding interface

The RT loop of the p120RasGAP SH3 domain appears critical to the interaction and makes contact along the length of the loop (Fig. 2b). The most notable feature of the RT loop binding is the direct interaction with the catalytic arginine finger residue Arg-1114 in DLC1 RhoGAP, located in the αA-αA1 loop. Specifically, Arg-1114 forms a salt bridge with the sidechain of Glu-298 and a hydrogen bond with the sidechain of Tyr-290, and additional van der Waals interactions with Val-293 in the RT loop and Trp-317 at the start of β3. At the tip of the RT loop, Asp-295 makes a salt bridge to Arg-1155 at the end of αB, the carbonyl of Thr-296 hydrogen bonds to Thr-1238 in the FG loop, and the sidechain of Asp-297 hydrogen bonds with the sidechain of Asn-1266 in αG (Fig. 2b, inset). Nearby, there are additional van der Waals interactions contributed by Val-1227, Pro-1231, and Leu-1236. In addition to its RT loop interactions, the SH3 domain n-Src loop residues Glu-314, Asp-315 and Trp-317 interact with DLC1 residues Thr-1221, Pro-1222 and Thr-1223, with a hydrogen bond forming between the sidechains of Asp-315 and Thr-1223 (Fig. 2c). Also, the β2-β3-β4 strands of p120RasGAP SH3 domain bind DLC1 along one face of αG (Fig. 2d). At the center of this interface are Leu-1263 and Leu-1267 of DLC1, which bind the hydrophobic region created by SH3 Trp-319, Leu-313 and Asn-311. Gln-1260 and Gln-1271 bracket this interface, with Gln-1260 forming a hydrogen bond to Gln-328. Taken together, there are extensive interactions between the p120RasGAP SH3 domain and DLC1 RhoGAP domain to create a large interface.

### SH3 domain binding site overlaps RhoA binding site on DLC1

**Fig. 3:**
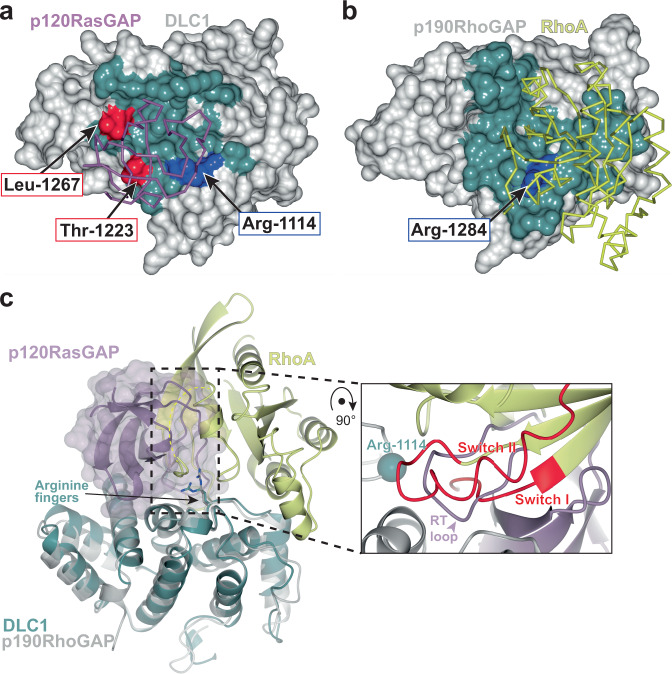
Comparison of the RhoGAP binding sites for RhoA and p120RasGAP SH3 domain. **a** Surface of DLC1 RhoGAP domain with p120RasGAP SH3 domain binding site highlighted in blue. The Arginine finger Arg-1114 is shown in blue and labeled, and two surface residues mutated in this study (Thr-1223 and Leu-1267) are shown in red. **b** Surface of p190RhoGAP with RhoA binding site highlighted in teal (PDB ID: 5IRC^7^). The Arginine finger Arg-1284 is shown in red and labeled. Views in A and B are similar after superposition of the RhoGAP domains. Interface residues in (A) and (B) are defined by CCP4mg^84^ and is based on buried surface. **c** Structure of RhoA in complex with p190RhoGAP (PDB ID: 5IRC, gray and yellow) superposed with p120RasGAP SH3 in complex with DLC1 (green and purple). Superposition on the RhoGAP domains. The p120RasGAP SH3 domain (purple) is shown as ribbon and transparent surface to highlight the overlap in binding sites. Inset: the SH3 domain RT loop is a close mimic of the switch I and switch II regions of the active (GTP-bound) conformation of RhoA. The location of the arginine finger Arg-1114 Ca is indicated as a sphere.

Previous work demonstrated that the p120RasGAP SH3 domain inhibits RhoGAP activity of DLC1^32,33^. We asked whether this inhibition is due to interference with the Rho GTPase binding site. There is currently no experimentally determined three-dimensional structure of the complex between the RhoGAP domain of any DLC protein and a GTPase. However, a consensus interface between RhoGAP and Rho GTPases has been postulated^7,45^ based on highly conserved features observed in structures of Rho GTPase-RhoGAP complexes (e.g., RhoA-p50RhoGAP^40^ and RhoA-p190RhoGAP-A^7^). Because the RhoGAP domain of DLC1 is highly similar to other RhoGAPs (e.g., RMSD 1.6 Å over 179 equivalent Cα positions when compared to p190RhoGAP, PDB ID: 5IRC^7^), we can compare the SH3 binding site with the predicted interface between DLC1 and RhoA: the RhoA binding site on DLC1 is predicted to be highly similar to that of p190RhoGAP (Fig. 3a). Comparison of the Rho binding site on p190RhoGAP with our experimentally determined SH3 binding site on DLC1 reveals that these sites partially overlap (Fig. 3b). This strongly suggests that DLC1 RhoGAP cannot bind both RhoA and SH3 simultaneously, but instead supports that p120RasGAP SH3 can directly compete with RhoA for DLC1 binding. We, therefore, hypothesize that SH3 inhibits the DLC1 RhoGAP activity by direct competition with RhoA for interaction with DLC1.

In more detail, the location of the SH3 interaction site suggests functional importance. It is well established that RhoGAP domains bind the GTP-loaded (active) conformation of Rho GTPases at a binding site that includes the switch I and switch II regions^4,40^. Our analysis suggests that the SH3 domain RT loop overlaps with the RhoA switch I and switch II regions, indicating that the most extensive region of SH3 domain recognition corresponds with the most functionally relevant region of RhoA recognition (Fig. 3c). Notably, the arginine finger of the DLC1 RhoGAP domain is critical to both RhoA and SH3 binding (Fig. 3c).

### SH3 domain inhibits DLC1 RhoGAP activity in vitro

To validate the molecular basis of p120RasGAP inhibition of DLC1 we conducted biochemical GTPase activity assays. We established an in vitro RhoA GTPase activity assay that utilizes the malachite green colorimetric reagent to directly monitor production of inorganic phosphate^46^. Similar to assays presented for Ras^47^, this method has the advantage of directly monitoring the production of inorganic phosphate by hydrolysis without the need for a fluorescently tagged GTP analog. This assay also avoids any steric interference by a fluorescent moiety. DLC1 displays preference for RhoA, RhoB and RhoC over the other Rho GTPase family members^7^; therefore, to assess GAP activity we assessed DLC1 activity on RhoA hydrolysis. Additionally, to allow for nucleotide exchange and RhoA to perform multiple hydrolysis cycles we performed these assays in the presence of both EDTA and magnesium.

**Fig. 4:**
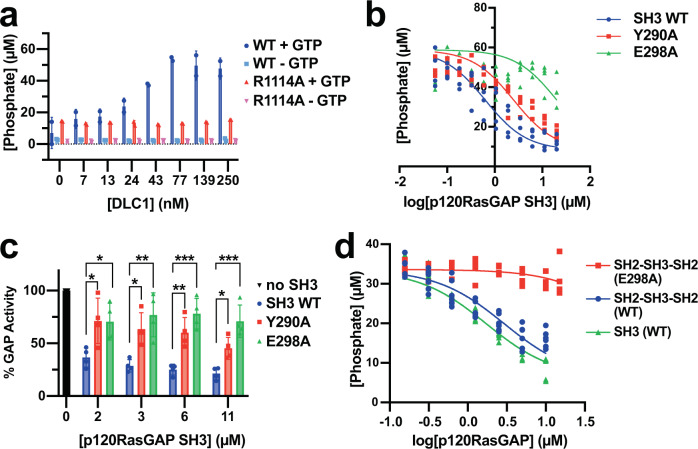
Biochemical assessment of p120RasGAP SH3 domain inhibition of DLC1 RhoGAP activity. **a** DLC1 RhoGAP wild-type (WT, blue and cyan) but not R1114A mutant (red and pink) stimulates RhoA GTPase activity. The concentration of phosphate generated (μM) with added GTP (+GTP) or without GTP (−GTP) is shown. Data are shown as mean values (bars) +/− SD (error bars), and individual measurements are plotted (dots, *n* = 2). **b** Titration of p120RasGAP SH3 domain (0–20 μM) wild type (WT, blue) and structurally defined mutants Y290A (red) and E298A (green) to inhibit DLC1 RhoGAP activity. The phosphate generated (in μM) is plotted against the log10 of SH3 concentration (μM), IC_50_ values were calculated by nonlinear regression (lines), and individual measurements are plotted (dots, *n* = 4). **c** Representative SH3 domain concentrations from (**b**) (2, 3, 6, and 10 μM). % GAP activity is normalized to the maximum phosphate generated by DLC1 stimulated RhoA (black bar, 100%). Data are mean values (bar graph) +/− SD (error bars), and individual measurements are plotted (dots, n = 4). *P* values: WT vs Y290A: 2 μM SH3: *P* = 0.0414; 3 μM SH3: *P* = 0.0263; 6 μM SH3: *P* = 0.0070; 11 μM SH3: *P* = 0.0346; WT vs E298A: 2 μM SH3: *P* = 0.0455; 3 μM SH3: *P* = 0.0041; 6 μM SH3: *P* = 0.0004; 11 μM SH3: *P* = 0.0004. **d** Inhibition of DLC1 RhoGAP activity by p120RasGAP SH2-SH3-SH2 wild type (blue) or E298A mutant (red), and SH3 domain (green). The phosphate signal (μM) is plotted against the log10 of p120RasGAP concentration. IC_50_ values were calculated by nonlinear regression (lines), and individual measurements are plotted (dots, *n* = 7). In **b**, **d**, IC_50_ values are calculated by nonlinear regression in GraphPad Prism using the One site–fit logIC50 model, and are reported in Table 2. In **c**, *P* values are calculated in GraphPad Prism using ordinary one-way ANOVA analysis with Tukey’s multiple comparison test. Significant differences are based on *P* values as indicated:* *P* = 0.01 to 0.05;** *P* = 0.001 to 0.01;*** *P* = 0.0001 to 0.001. Source data are available as a Source Data file.

Our initial assays demonstrated that the inefficient intrinsic GTP hydrolysis by purified RhoA is catalytically enhanced in the presence of increasing concentrations of DLC1 RhoGAP domain (Fig. 4a). In contrast, the Arginine-finger mutant R1114A of DLC1 RhoGAP does not stimulate hydrolysis (Fig. 4a). We next assessed the inhibition of DLC1 RhoGAP activity by the p120RasGAP SH3 domain. We performed a titration of SH3 domain from 0 to 20 μM in an assay containing 5 μM RhoA and 0.05 μM DLC1 RhoGAP. We observe a dose-dependent inhibition of DLC1 RhoGAP activity by the SH3 domain (Fig. 4b). Plotting the phosphate generation versus log p120RasGAP SH3 concentration allowed us to calculate an IC_50_ of 0.6 μM (95% confidence interval 0.48–0.83 μM) (Table 2). Although not a direct measurement of affinity, this calculated IC_50_ value is in remarkably close agreement with the previously reported dissociation constant (*K*_*d*_) of 0.6 μM measured by isothermal titration calorimetry and the *K*_*i*_ of 0.6 μM determined by kinetics^33^.

**Table 2:** IC_50_ calculation for p120RasGAP inhibition of DLC1 RhoGAP activity

SH3 (Fig. 4b)	IC_50_ (μM)	95% CI (μM)	*R*^2^
WT	0.63	0.48 to 0.83	0.8
Y290A	2.5	1.9 to 3.3	0.7
E298A	20	12 to 36	−1.6
**SH2-SH3-SH2** **(Fig.** 4d**)**			
WT	2.9	2.4 to 3.6	0.8
E298A	115	67 to 310	0.2
SH3 WT	1.7	1.5 to 2.0	0.93

Top: p120RasGAP SH3 domain and mutants. The Y290A and E298A mutants have increased IC_50_ values relative to the wildtype. Negative *R*^2^ value for E298A indicates ineffective inhibition. Bottom: p120RasGAP SH2-SH3-SH2 domains compared to SH3 domain. All values are calculated using GraphPad (GraphPad Prism Version 9).

The co-crystal structure of DLC1 RhoGAP domain in complex with p120RasGAP SH3 domain reveals a direct interaction of the Arginine finger Arg-1114 with SH3 residues Tyr-290 and Glu-298 (Fig. 2b). To test the importance of this interface, we generated single point alanine mutant SH3 domains Y290A and E298A and tested their ability to inhibit DLC1RhoGAP in a dose-dependent manner. Both mutant SH3 domains demonstrate reduced inhibition of DLC1 (Fig. 4b, c) with calculated IC_50_ values 4-fold and over 30-fold lower for the Y290A and E298A mutants, respectively (Table 2). These inhibition assays therefore support the structural observation that the SH3 domain interacts directly with the DLC1 RhoGAP domain via these residues.

Since the p120RasGAP SH3 domain is closely flanked by SH2 domains (Fig. 1a), we next asked whether inhibition of DLC1 by the SH3 domain is affected by these flanking SH2 domains by titrating increasing amounts of p120RasGAP SH2-SH3-SH2 or SH3 domain protein and monitoring the phosphate release by the BIOMOL green assay (Enzo Life Sciences) which is complementary to the Malachite green assay used above. We plotted the phosphate release versus p120RasGAP concentration (Fig. 4d) and determine similar IC_50_ values of the SH2-SH3-SH2 domain and SH3 domain of 2.9 and 1.7 μM, respectively (Table 2). We also observe that mutation of the crystallographically-defined interface residue Glu-298 (Fig. 2b) in the context of the SH2-SH3-SH2 protein abrogates the inhibition (Fig. 4d and Table 2). Thus, the presence of the flanking SH2 domains does not strongly alter the inhibitory activity of the SH3 domain in p120RasGAP.

### DLC1 mutants are not inhibited by p120RasGAP

**Fig. 5:**
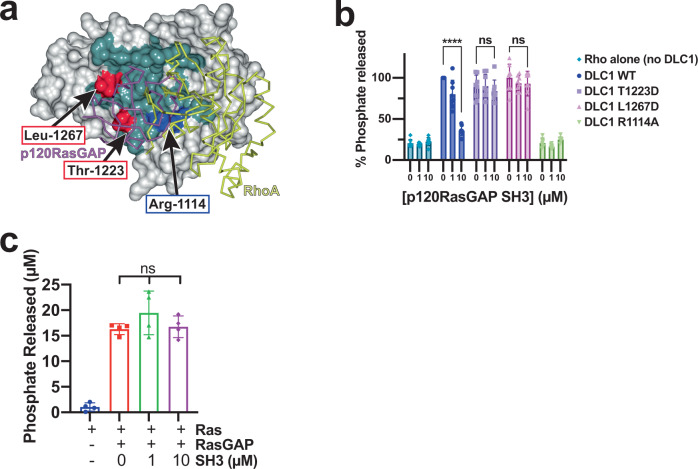
DLC1 RhoGAP mutants and p120RasGAP are not inhibited by SH3 domain. **a** Surface representation of DLC1 illustrating the differences in the footprint of binding by p120RasGAP SH3 domain (purple) and RhoA (yellow, predicted), with residues that bind p120RasGAP colored teal. DLC1 residues mutated in this study are labeled. **b** RhoGAP activity of structurally-defined DLC1 mutants T1223D and L1267D are not inhibited by p120RasGAP SH3 domain. RhoA alone (teal) or RhoA plus the arginine finger mutant DLC1 R1114A (green) are included. Signal is plotted as % phosphate generated, normalized to activity of RhoA plus wild-type DLC1 (blue, 0 μM SH3). Data are presented as mean values (bars) +/− SD (error bars), and individual measurements are plotted (dots, *n* = 8). *P* values: WT 0 μM versus 10 μM SH3, *P* < 0.0001; T1223D 0 μM versus 10 μM SH3, *P* = 0.7747; L1267D 0 μM versus 10 μM SH3, *P* = 0.5663. **c** GTP hydrolysis by Ras (blue) is stimulated by p120RasRasGAP RasGAP domain (red) but is not inhibited by 1 μM (green) or 10 μM (purple) p120RasGAP SH3 domain. Data are presented as mean values (bars) +/− SD (error bars), and individual measurements are plotted (dots, *n* = 4). *P* values are: 0 μM versus 1 μM SH3: 0.3148; 0 μM versus 10 μM SH3: 0.9944; 1 μM versus 10 μM SH3: 0.4329. In **b**, **c** *P* values were calculated by ordinary one-way ANOVA analysis with Tukey’s multiple comparison test in GraphPad Prism. Significant differences are based on *P* values as indicated: ns: *P* ≥ 0.05; **** *P* < 0.0001. Source data are available as a Source Data file.

Our structural analysis of the complex between DLC1 and p120RasGAP, and comparison with other RhoGAP co-crystal structures in complex with Rho family small GTPases (Fig. 3a, b) suggest different binding footprints for p120RasGAP SH3 domain when compared to small GTPases. On closer inspection, in αF of DLC1 the Thr-1223 sidechain forms a hydrogen bond to Asp-315 of the p120RasGAP SH3 n-Src loop and contacts Asp-314, Asp-315 and Trp-317 by van der Waals interactions (Fig. 2c). Similarly, in αG of DLC1 the Leu-1267 sidechain of is at the center of a hydrophobic patch and interacts with SH3 domain residues Asn-311, Leu-313 and Trp-319 (Fig. 2d). Comparison of the locations of these residues with the expected small GTPase binding footprint suggests that their point mutation may result in disruption of p120RasGAP binding but not of RhoA (Figs. 3a, b and 5a) thus resulting in a loss of inhibition by SH3 but leaving the RhoGAP activity intact. We, therefore, introduced aspartate mutations at these positions, T1223D and L1267D, and assessed GTP hydrolysis in the malachite green assay. We find that suppression of RhoGAP activity by p120RasGAP SH3 domain is lost for both T1223D and L1267D mutant DLC1 RhoGAP proteins (Fig. 5b), although both mutant DLC1 proteins maintain their GAP activity toward RhoA in the absence of SH3 domain (Fig. 5b, 0 μM SH3 samples). These data reflect that these mutants interrupt the binding of p120RasGAP SH3 domain but do not impact activity towards RhoA. The co-crystal structure of DLC1 RhoGAP domain in complex with p120RasGAP domain, therefore, describes a binding site that overlaps with the Rho binding site, but that can be manipulated to selectively disrupt RhoGAP inhibition by p120RasGAP.

Lastly, we asked whether the p120RasGAP SH3 domain can inhibit its own RasGAP domain by measuring GTP hydrolysis by H-Ras in the presence of the isolated RasGAP and SH3 domains, and find that RasGAP, which stimulates the intrinsic hydrolysis of H-Ras, is not inhibited by excess amounts of SH3 domain (Fig. 5c). Thus, p120RasGAP does not employ an SH3/RasGAP interface as a mode of autoinhibition.

### p120RasGAP has an atypical SH3 domain that binds RhoGAP domain

**Fig. 6:**
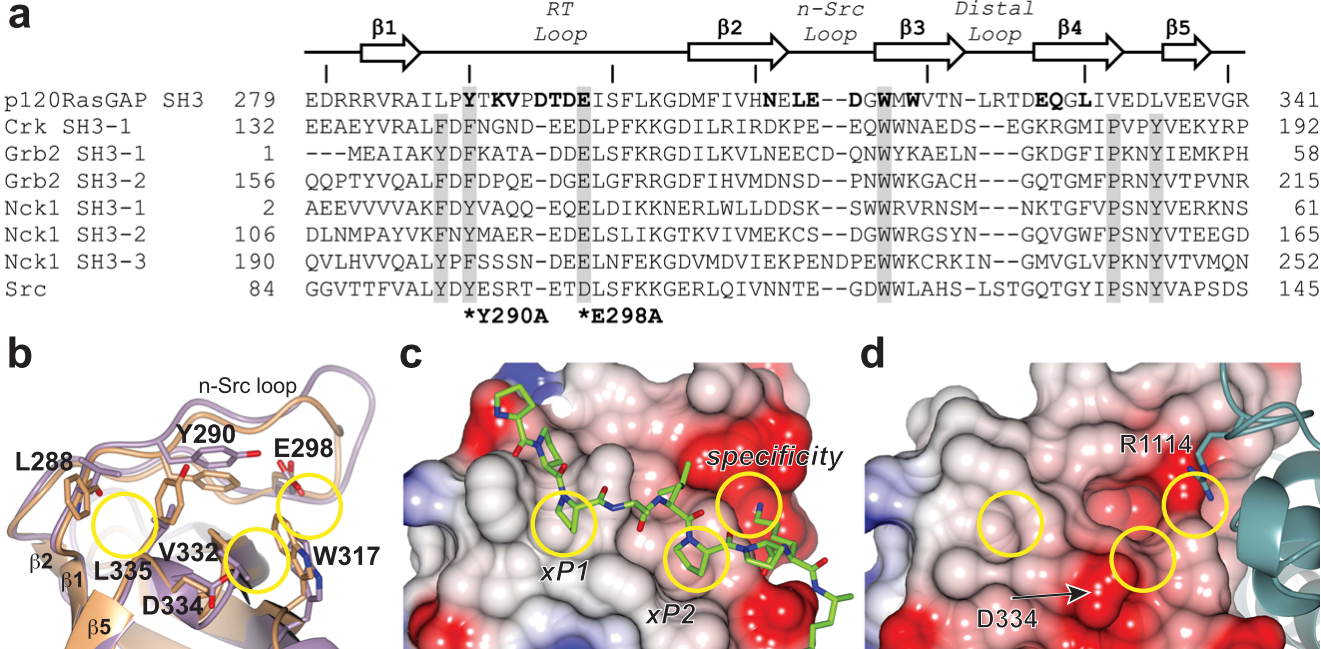
p120RasGAP harbors an unusual SH3 domain. **a** Structure-based sequence alignment (Dali server) of SH3 domain of p120RasGAP with SH3 domains of human Crk (Uniprot ID: P46108), Grb2 (Uniprot ID: P62993), Nck1 (Uniprot ID: P16333) and Src (Uniprot ID: P12931). SH3-1, -2 or -3 indicates the specific SH3 domain within a protein containing multiple SH3 domains. Residues in p120RasGAP that interact with the RhoGAP of DLC1 are highlighted in bold. Residues in canonical SH3 domains that form the conserved xP binding pockets (xP1 and xP2) and specificity pocket are shaded in gray. Positions of residues mutated in this study - Tyr-290 and Glu-298 - are marked (*) and labeled. In a previous study, only p120RasGAP SH3 domain could inhibit DLC1 RhoGAP activity among these SH3 domains^33^. Every tenth residue in p120RasGAP is marked with a line. **b** Residues lining the xP1 and xP2 binding pockets and the specificity pockets of p120RasGAP (purple) and Crk SH3-1 domain (orange) (PDB ID: 1CKA^70^). Selected residues of p120RasGAP are labeled. **c**, **d** Electrostatic surface potential of (**c**) Crk SH3 domain in complex with a RapGEF1 peptide shown in stick format (green), **d** p120RasGAP SH3 domain in complex with DLC1 RhoGAP domain shown in ribbon format (teal). xP1, xP2 and specificity pocket locations are circled as in (**b**).

The SH3 domain of p120RasGAP is unusual compared to canonical SH3 domains, and its interaction with DLC1 RhoGAP domain may help explain its non-canonical features. Canonical SH3 domains exhibit canonical binding activity toward left-handed PxxP (x is any amino acid) polyproline II (PPII) helical peptides^48–50^. These SH3 domains contain three binding pockets on their surfaces between the RT and n-Src loops: two shallow xP dipeptide binding sites, and a third acidic specificity site that binds a positively charged peptide residue and helps determine the directionality of peptide binding^31,35,51,52^. The two xP sites (xP1 and xP2) are comprised of aromatic residues in the RT loop, β4, and 3/10 loop and are conserved in most PxxP-binding SH3 domains (Fig. 6a–c).

Previous crystal structures of p120RasGAP SH3 domain revealed divergent structural features at the putative xP binding sites compared to canonical SH3 domains^43^, and our co-crystal structure confirms this divergence. These structures suggest that p120RasGAP may not bind canonical polyproline-containing ligands since Leu-288 and Leu-335 replace aromatic residues in xP1, and Val-332 replaces a typical Proline in xP2 (Fig. 6a, b). Additionally, we observe that Asp-334, which is often an uncharged Proline or Asparagine residue in canonical SH3 domains (Fig. 6a, b), partially obscures the xP2 site (Fig. 6b, d). In support of these structural analyses that suggest a lack of a canonical binding site, no polyproline-containing ligand has been identified for p120RasGAP SH3 domain^30,31^. Thus, if a yet-unidentified atypical peptide ligand for p120RasGAP SH3 exists, the mode of binding would be expected to diverge significantly from typical SH3-peptide interactions.

Nonetheless, despite the lack of conserved xP1 and xP2 sites, the p120RasGAP SH3 domain contains a conserved acidic residue in the RT loop, Glu-298, that typically forms the specificity pocket in SH3 domains (Fig. 6a, d)^31,35^. Interestingly, in the co-crystal structure of p120RasGAP with DLC1 we find that Glu-298, together with Tyr-290, which is a conserved aromatic residue in the xP2 binding pocket, directly coordinate the arginine finger residue Arg-1114 of RhoGAP (Fig. 2b, d). Trp-317, which can contribute to the xP2 pocket, specificity pocket, or both^35^, also directly contacts DLC1. This potentially suggests a degradation of polyproline binding function, but retention and repurposing of the specificity pocket to recognize the critical arginine finger of DLC RhoGAP proteins.

## Discussion

DLC1 is a potent tumor suppressor which is deleted in many human tumors^10,53^. Its loss is associated with enhancement of both cell proliferation and migration, while its overexpression leads to reduction of cell growth and motility^9,54,55^, suggesting that intricate control of its activity is required for normal cell growth. Autoregulation of DLC1 involving the SAM and/or SMART domains has been demonstrated^9,18^, and both localization^56^ and phosphorylation^57–59^ may be similarly involved. Importantly, higher order protein complexes form^24^, and binding of partner proteins is implicated in DLC1 regulation (reviewed in ref. 60). It is the direct regulation of DLC1 by p120RasGAP that we assess at the molecular level in this study. We determined the co-crystal structure of the SH3 domain of p120RasGAP in complex with the RhoGAP domain of DLC1. The structure demonstrates a previously underappreciated role of SH3 domains as direct modulators RhoGAP proteins and illustrates a mode of binding that is competitive with the interactions of the RhoGAP domain with small GTPases. Our structure and analysis highlight several features that are distinct for the recognition of DLC1 by p120RhoGAP, provide insight into DLC1 regulation, and raise the possibility of alternate regulation mechanisms for small GTPases.

The inhibition of DLC1 RhoGAP activity by p120RasGAP SH3 domain demonstrates a clear and direct mechanism by which crosstalk between the Rho and Ras pathways can occur via interaction of their cognate regulatory proteins^33^. The multiple modular domains in both the RhoGAP and RasGAP protein families have been shown to act as scaffolds that promote pathway crosstalk^8,61^, and the interaction of DLC1 with p120RasGAP provides one such intersection point that may affect the balance of Rho vs Ras signaling. In general, crosstalk between these critical small GTPase pathways can play key roles in modulating cell proliferation, spreading, migration and growth, and in cancer, and multiple nodes have been identified that impact both Ras and Rho pathways (reviewed in refs. 62, 63). Our co-crystal structure demonstrates a molecular level basis for direct Ras pathway impingement on Rho signaling cascades. To achieve this, the binding site of the p120RasGAP SH3 domain and its recognition of DLC1 is unique compared to other known typical and atypical SH3 binding sites and RhoGAP regulation mechanisms.

Furthermore, the question remains whether the balance between the Ras and Rho pathways via p120RasGAP may be dysregulated in transformed cells that lack DLC1. DLC1 is an important tumor suppressor that is mutated in many cancers, nearly as frequently as the commonly known p53 tumor suppressor^53^. Targeting of DLC1 activity via p120RasGAP in cancer cells has been the subject of at least one study^64^; however, future studies will be necessary to help better define the spatio-temporal nature of the interaction between these proteins and whether cellular transformation by loss of DLC1 involves Ras signaling via p120RasGAP.

High sequence similarity between DLC1 and its family members DLC2 and DLC3 suggests similar mechanisms of regulation across the family: the full-length sequences display identities of 54 and 47%, for DLC1 vs DLC2 and DLC1 vs DLC3, respectively, and RhoGAP domain sequence identities of 81 and 70%, respectively. There are few sequence insertions in the RhoGAP domains, and the arginine finger residues are in a conserved location (Supplementary Fig. 3). Nonetheless, previous studies found that DLC2 and DLC3 are suppressed to a lesser extent by p120RasGAP^33^, implicating that DLC1 is unique in its ability to be potently inhibited by the SH3 of p120RasGAP. Analysis of the conservation of residues of DLC1 that bind p120RasGAP suggests that these are only partially conserved in DLC2 and DLC3 (Supplementary Fig. 3). Importantly, the two residues we mutated to abolish binding to p120RasGAP SH3, Thr-1223 and Leu-1267, are not well conserved in DLC2 (Met-808 and Leu-851) and DLC3 (Gly-716 and Met-760). Previous studies and structural analysis together imply that the modulation of RhoGAP activity by p120RasGAP may be favored toward DLC1, suggesting isoform-specific effects on signaling cascades downstream of different cellular stimuli. Thus, DLC1 may be unique compared to its related family members and likely among the large RhoGAP family in its binding and regulation by the SH3 domain of p120RasGAP.

Many RhoGAP proteins are multidomain proteins, and regulation of their RhoGAP activities may involve intramolecular inhibition via direct interaction *in cis* of these modular domains with the RhoGAP domain^65^. For example, it has been postulated that in the Graf RhoGAP family including Graf1 and oligophrenin, the N-terminal BAR (Bin/amphiphysin/Rvs) domain autoinhibits the RhoGAP activity by binding and masking the RhoGAP active site^66,67^. Similarly, p50RhoGAP is thought to be autoinhibited by binding of its BCH domain to the RhoGAP active site^68^. However, molecular level details of these interactions are currently unobserved. Additionally, RhoGAPs may be inhibited in trans by a binding partner, like DLC1 by p120RasGAP, but the details of regulation are unknown. Our structure, therefore, provides a molecular level observation of inhibition of a RhoGAP domain by direct masking of the catalytic arginine finger. Whether other RhoGAP proteins are regulated by masking of the arginine finger by direct protein binding remains to be revealed.

**Fig. 7:**
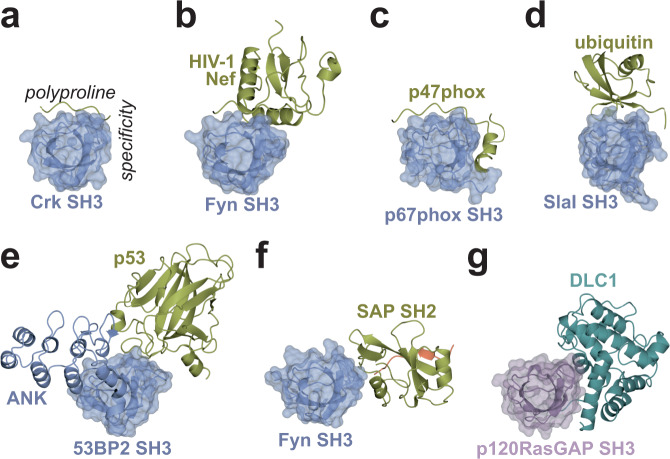
Comparison of atypical SH3 binding partners. Examples of atypical SH3 domain complexes that bind partner proteins outside of the canonical polyproline site. In all panels, the SH3 domain (shown as light blue ribbon and partially transparent surface in (**a**) thru (**f**) orientation and scale is identical, with the polyproline site on top and the specificity pocket is on the right hand side as labeled in (**a**). All binding partners in panels (**a**) thru (**f**) are shown as ribbon diagrams and colored gold. **a** Crk/peptide (to illustrate canonical binding; PDB ID: 1CKA^70^). **b** Fyn SH3 bound to the HIV Nef protein (PDB ID: 1EFN^72^). **c** p67phox SH3 bound to p47phox (PDB ID: 1K4U^71^). **d** Yeast SlaI SH3 domain bound to ubiquitin (PDB ID: 2JT4^73^). **e** 53BP2 SH3 domain interacting with its own ANK repeat region and bound to p53 (gold) (PDB ID: 1YCS^75^). **f** Fyn SH3 domain bound to SAP SH2 domain which is bound the Slam peptide (orange) (PDB ID: 1M27^34^). **g** DLC1 RhoGAP (teal) with p120RasGAP SH3 (purple).

Our structure also represents a noncanonical role for an SH3 domain binding site: to regulate a RhoGAP protein. Previous studies have demonstrated that SH3 domains can mediate non-canonical interactions (reviewed in refs. 31 and 35, 69). These atypical interactions can involve secondary structure elements that extend beyond the canonical polyproline peptide (e.g., Crk, Fig. 7a, PDB ID: 1CKA^70^), or as observed for the p67phox SH3 binding p47phox via both polyproline and a unique helix-turn-helix structure that binds the specificity site (PDB ID: 1K4U^71^, Fig. 7b). Alternatively, the polyproline motif can present as part of a folded domain, as observed in the HIV-1 Nef protein core domain bound to the Fyn SH3 domain (PDB ID: 1EFN^72^, Fig. 7c). Additionally, there are SH3 interactions that are devoid of typical polyproline peptide contacts and instead are mediated solely by tertiary contacts with other protein domains. Examples of these protein-protein interactions include the SH3 domain of SlaI (yeast), CIN85 and amphiphysin, which bind ubiquitin at the polyproline binding sites (PDB ID: 2JT4^73,74^, Fig. 7d), the SH3 domain of 53BP2, whose polyproline site is partially masked by its own ANK repeat region, binding the core domain of p53 (PDB ID: 1YCS^75^, Fig. 7e), and the Fyn SH3 domain which binds the SAP SH2 domain near the specificity pocket but away from the polyproline binding site (PDB ID: 1M27^34^, Fig. 7f). The atypical selectivity of these SH3 domains is generally associated with the variable lengths and sequences of n-Src and RT loops in the SH3 domain family^31^. We now add to this growing list the p120RasGAP SH3 domain, which uses an extended surface that includes the specificity site to bind to DLC1 RhoGAP; interestingly, the recognition and cradling of an SH3 domain by a catalytic domain is thus far unique to DLC1 and p120RasGAP (Fig. 7g).

It remains to be seen if other SH3 domains can directly regulate RhoGAP activity, but a previous study suggested that the SH3 domains of Src, Crk, and Nck do not act as inhibitors of DLC1 activity^33^. Further work will be needed to identify other potential SH3-RhoGAP regulatory pairs. Interestingly, an SH3 domain in the RacGEF Asef binds and inhibits its Dbl homology domain and blocks the Rac1 binding site (PDB ID: 2DX1^76^), and this regulation mechanism may be shared among other SH3-domain-containing GEFs like collybistin I and intersectin-1. Thus, the SH3 domain fold may play an expanded role in the regulation of Rho family GTPase pathways.

Molecular modeling of protein structures is becoming more important. Deep learning models of the molecular structure of DLC1 RhoGAP domain by Alphafold (AF-Q96QB1-F1-model_v2.pdb^77^), while correctly predicting the overall fold have not adequately predicted the fine details of the structure. Alphafold presents helix αG in a position that is slightly extended away from the RhoGAP core, similar to other RhoGAP structures. In contrast, both experimentally determined crystal structures of DLC1 RhoGAP domain (our co-crystal structure here, and the previously unpublished apo structure (PDB ID: 3KUQ^39^)) reveal that this helix is packed closer against the RhoGAP domain. This compaction allows interaction with the SH3 domain, whereas the protruded position it creates steric clashes with the SH3 domain. (Supplementary Fig. 4). Perhaps future deep learning models might help reveal additional SH3-RhoGAP binding partners based on structural similarities with DLC1. Similarly, a previously reported triple mutant p120RasGAP SH3 domain (N311R/L313A/W319G) was shown to impair both interaction with and inhibition of DLC1RhoGAP^33^. These mutants were based on a PatchDock docking model and comparison with the co-crystal structure reveals an approximately 180° rotation in the SH3 domain, explaining why this previous study did not achieve full loss of inhibition by point mutagenesis. While molecular modeling is advancing, experimental studies remain paramount.

In summary, the co-crystal structure of DLC1 RhoGAP domain in complex with the SH3 domain of p120RasGAP reveals a mechanism by which Ras and Rho pathways can crosstalk, and a previously unobserved molecular mechanism by which SH3 domains impinge on GTPase signaling cascades.

## Methods

### Protein expression

A pET28-MHL expression plasmid for the His_6_-tagged RhoGAP domain of human DLC1A (residues 1074–1283, Uniprot ID: Q96QB1 isoform 2) was a gift from Cheryl Arrowsmith (Addgene plasmid #25333; http://n2t.net/addgene:25333; RRID:Addgene_25333). cDNA encoding the SH3 domain of human p120RasGAP (residues 281–341, Uniprot ID: P20936) was inserted into pGEX-6p1 vector (Cytiva) for expression as a GST-fusion protein, and the SH2-SH3-SH2 domains (residues 172–443) or RasGAP domain (residues 714–1047) were inserted into a modified pET vector containing a His_6_-tag and TEV cleavage site. Wild-type human RhoA cDNA (Uniprot ID: P61586) was inserted into pGEX-6p1 (Cytiva) for expression as a GST-tagged protein. A stop codon was introduced after residue 181 by Quikchange Mutagenesis (Agilent) to encode a truncated protein (residues 1–181) lacking a portion of the C-terminal hypervariable region to avoid modification and membrane localization. Site directed mutagenesis of SH3 (Y290A, E398A) and DLC1 (R1114A, T1223D, L1267D) was also performed with QuikChange Mutagenesis (Agilent). Human H-Ras cDNA encoding residues 1–167 (UniProt ID: P01112) was subcloned into a modified pET vector for expression as N-terminally His_6_-tagged protein. All recombinant protein expression was performed in BL21(DE3) or Rosetta(DE3) *E. coli* cultures by induction with 0.2 mM isopropyl β-d-thiogalactopyranoside at cell density OD_600_ = 0.6–0.8 for 18 h at 18 °C and harvested by centrifugation.

### Protein purification

Cells expressing His_6_-DLC1, His_6_-H-Ras, His_6_-p120RasGAP SH2-SH3-SH2 or GAP domain protein were resuspended in lysis buffer containing 50 mM HEPES pH 7.3, 500 mM NaCl and 20 mM imidazole, and cells expressing GST-SH3 or GST-RhoA were resuspended in lysis buffer containing 20 mM Tris pH 7.4, 150 mM NaCl and 1 mM dithiothreitol. Cells were lysed by three freeze/thaw cycles in the presence of 0.1 mg/ml lysozyme followed by sonication (4 min total, 25% amplitude, QSonica). Total cellular lysate was clarified by centrifugation at 5000 × *g* at 4 °C for 1 h. His_6_-tagged DLC1 RhoGAP, HRas, p120RasGAP SH2-SH3-SH2 or GAP domain proteins were purified by applying clarified lysate to nickel-nitrilotriacetic acid (Ni-NTA) agarose beads (Qiagen) for 1 h, which were then washed with lysis buffer prior to step-wise protein elution with lysis buffer containing increasing concentrations of imidazole (40 mM–100 mM–250 mM–500 mM). Fractions containing protein of interest were pooled and applied to size exclusion chromatography (Superdex75 Increase) column equilibrated in buffer containing 20 mM Tris pH 8.0, 150 mM NaCl. The final protein was concentrated in a spin concentrator (Amicon, MilliporeSigma). H-Ras and p120RasGAP GAP domain proteins were additionally purified by anion exchange chromatography (Mono Q 5/50 GL Cytiva) in 20 mM Tris pH 8 and eluted by a gradient of NaCl from 0.05 to 1 M. DLC1 RhoGAP was used in its His_6_-tagged form for both crystallization and biochemical assays; likewise, His_6_-HRas and His_6_-p120RasGAP GAP domain were used in biochemical assays.

GST-SH3 or GST-RhoA proteins were purified by affinity chromatography by incubating clarified lysate with Glutathione Sepharose beads (Cytiva) in a gravity flow column at 4 °C for 2 h. Beads were washed with 20 column volumes of lysis buffer, and resuspended in lysis buffer at a 1:1 bead:buffer ratio by volume. GST-fusion proteins were proteolytically cleaved by addition of GST-Prescision protease on-bead. Tag-free SH3 or RhoA proteins were collected as flow-through and further purified over size exclusion chromatography (Superdex 75 prep grade, Cytiva) in buffer containing 20 mM Tris pH 8.0 and 150 mM NaCl. Proteins were concentrated in spin concentrators (Amicon, MilliporeSigma).

For co-crystallization protein samples, cells expressing His_6_-DLC1 RhoGAP and GST-p120RasGAP SH3 were mixed prior to lysis, which was carried out as described above. The complex, which formed in solution, was purified over glutathione affinity chromatography (Cytiva), liberated from beads by PreScission protease cleavage of the GST tag, and purified by size exclusion chromatography in 20 mM Tris pH 8.0, 150 mM NaCl to yield the protein complex and efficiently separate the excess p120RasGAP SH3 alone.

### X-ray crystallography

Crystallization trials were carried out with a protein sample mixture consisting of a copurified complex of DLC1 RhoGAP with p120RasGAP SH3 at 18 mg/ml. Screen setup was performed using a TTP Labtech Mosquito in sitting-drop vapor diffusion setups at ambient temperature (∼22 °C). Initial co-crystals were obtained in PEG/Ion Screen (Hampton Research) condition B4, comprised of 0.2 M Magnesium nitrate, 20% PEG 3350, pH 5.9. Optimal crystals, which grew as rods with approximate dimension 100 μm × 20 μm × 20 μm, were obtained in 0.2 M Magnesium nitrate, 16.5% PEG 3350, in 4 μl hanging drops (2 μl of protein complex plus 2 μl reservoir solution) over 1 ml reservoir solution. Crystals were harvested and cryopreserved in reservoir solution supplemented with 20% ethylene glycol and flash cooled in liquid nitrogen.

X-ray diffraction data were collected from a single crystal at the Northeastern Collaborative Access Team (NECAT) Beamline 24-ID-E at Argonne National Laboratory Advanced Photon Source. X-ray data were processed and scaled in HKL2000^78^ to 3.2 Å resolution in spacegroup H3 with unit cell dimensions *a* = *b* = 143.7 Å, *c* = 152.8 Å, *α* = *β* = 90°, *γ* = 120°. XTriage analysis predicts four copies of the 1:1 complex per asymmetric unit with a solvent content of 46.5%.

The structure was solved by molecular replacement using Phenix Phaser^79^. Four copies of the DLC1 RhoGAP domain were placed using the previously unpublished DLC1A structure (PDB ID: 3KUQ^39^) as a search model, with final TFZ score of 34.5 and LLG 1831. Next, placement of two of four expected SH3 domains was achieved using the crystal structure of p120RasGAPSH3 domain (PDB ID: 2J05^43^) as search model with TFZ scores of 18.0 and 20.5; placement of the third and fourth copies failed. Each SH3 domain formed an identical interface with an individual DLC1RhoGAP copy in the asymmetric unit. Thus, the final two copies of SH3 were placed manually based on this interface with the remaining two DLC1RhoGAP copies and validated by refinement. Phenix Autobuild^80^, and manual building in Coot^81^, were performed. Refinement was carried out in Phenix^82^. A total of 1010 protein residues were modeled, with 5 N-terminal vector-derived residues in each DLC1 protein chain. All crystallography software was compiled by SBGrid^83^. Raw diffraction images have been deposited to the SBGRID Data Bank: 10.15785/SBGRID/876. Protein model coordinates and structure factors have been deposited to the Protein Data Bank with accession number 7TPB and prepared using PDB Extract version 3.27. Crystallization figures were generated in ccp4mg^84^. Protein interface calculations were performed on the PISA server (Protein Interfaces, Surfaces and Assemblies) at the European Bioinformatics Institute^44^, and structure-based alignments were run on the Dali server^85^.

### Malachite green assays

Hydrolysis of GTP by RhoA was monitored in an endpoint assay using the malachite green reagent (Malachite Green Kit (Sigma Millipore) or BIOMOL Green (Enzo Life Sciences)). All assays were performed with purified RhoA at 5 μM in assay buffer with a final composition of 20 mM Tris-HCl pH 8.0, 250 mM NaCl, 1 mM MgCl_2_ and 2 mM Ethylenediaminetetraacetic acid in final reaction volume of 80 μl. The reactions were initiated by addition of GTP at 50 μM. To monitor the effect of p120RasGAP SH3 domain, DLC1 RhoGAP protein at 0.05 μM and a titration series from 0 to 20 μM of wild-type or mutant SH3 domain protein were included. To assess the activity of wild-type and mutant DLC1, 0.1 μM DLC1 proteins were mixed with 0, 1, or 10 μM SH3 domain protein. The reactions were carried out at room temperature for 40 min in clear bottom 96-well plates (Corning) and terminated by addition of 20 μl of the malachite green working reagent, which was prepared as per the manufacturer’s instructions (Sigma Millipore), followed by color development for 30 min. A single absorbance reading at 620 nm for each sample was measured at room temperature in a Synergy H1 plate reader (BioTek) using Gen5 3.11 software. A standard phosphate curve with concentrations from 4 to 40 μM was used to generate a linear standard curve and was used to calculate the phosphate generated in each sample well. Reactions in the absence of added GTP were included to control for background phosphate content signal, which was found to be negligible. The IC_50_ of SH3 domain or SH2-SH3-SH2 domain inhibition were calculated in GraphPad Prism by plotting the mean phosphate signal (*n* = 4 or *n* = 7, respectively) versus log10 SH3 concentration using nonlinear regression using the model: One-Site - Fit LogIC50 and constraining the top and bottom values to the maximum (zero SH3 inhibition) and minimum (zero DLC1). Hydrolysis of GTP by Ras was performed similarly, using 5 μM purified H-Ras and 0.075 μM p120RasGAP GAP domain, in the presence of p120RasGAP SH3 domains at 0, 1, and 10 μM. For experiments using p120RasGAP SH2-SH3-SH2 domain, the hydrolysis reactions were performed using 200 μM GTP for 30 min, and color development for 20 min using the BIOMOL green malachite reagent (Enzo Life Sciences).

### Reporting summary

Further information on research design is available in the Nature Research Reporting Summary linked to this article.

## Supplementary information

Supplementary Information

Reporting Summary

## Data Availability

The data that support this study are available from the corresponding authors upon reasonable request. Coordinates have been deposited in the RSCB Protein Data Bank (PDB) under accession code 7TPB (DLC1 RhoGAP + p120RasGAP SH3). X-ray diffraction images are available online at SBGrid Data Bank: https://data.sbgrid.org/dataset/876/. Previously determined structures used in our analysis were obtained from the Protein Data Bank: 3KUQ (DLC1 RhoGAP domain), 1TX4 (Rho/RhoGAP complex), 2J05 (RasGAP SH3), 2J06 (RasGAP SH3), 4FSS (RasGAP SH3), 2GQI (RasGAP SH3), 2M51 (RasGAP SH3), 5IRC (RhoA/p190A RhoGAP domain complex), 1CKA (C-Crk SH3 domain), 1K4U (p67phox SH3 domain/p47phox tail complex), 1EFN (Fyn SH3 domain/HIV-1 Nef complex), 2JT4 (Sla1 SH3/Ubiquitin complex), 1YCS (P53-53BP2 complex), 1M27 (SAP/FynSH3/SLAM ternary complex), 2DX1 (Asef RhoGEF). The Alphafold model of DLC1 (AF-Q96QB1-F1-model_v2.pdb) was obtained from the Alphafold Structure Database: https://alphafold.ebi.ac.uk/files/AF-Q96QB1-F1-model_v2.pdb. The source data underlying Figs. 4a–d and 5b, c is provided as a Source Data file. Source data are provided with this paper.

## Source data

Source Data
