# Supplementary material for: SH3 domain regulation of RhoGAP activity: Crosstalk between p120RasGAP and DLC1 RhoGAP
Source: Nat Commun. 2022 Aug 15;13:4788. doi: 10.1038/s41467-022-32541-4 (PMC9378701; doi:10.1038/s41467-022-32541-4)
Supplement: Supplementary file 1 — Supplementary Information [file 41467_2022_32541_MOESM1_ESM.pdf]

SH3 domain regulation of RhoGAP activity: crosstalk between p120RasGAP and DLC1 RhoGAP

Jocelyn E. Chau [1], Kimberly J. Vish[1,2], Titus J. Boggon[1,2] and Amy L. Stiegler[2]*

1. Department of Molecular Biophysics and Biochemistry, Yale University, New Haven, CT, USA

2. Department of Pharmacology, Yale University, New Haven, CT, USA

* amy.stiegler@yale.edu

Supplementary Information

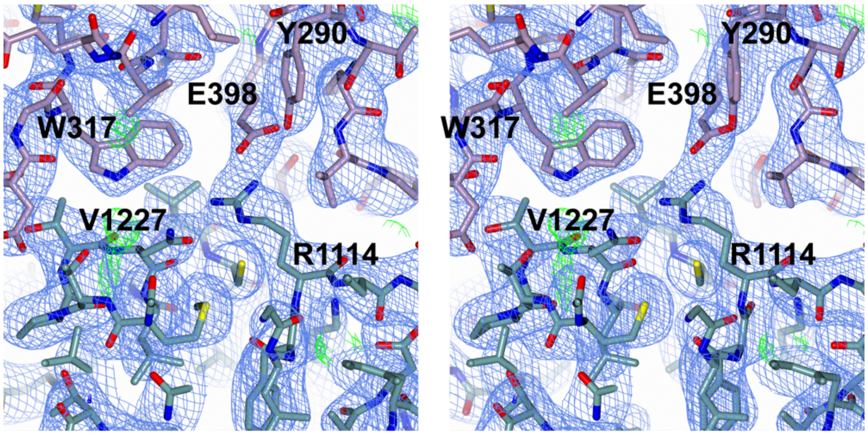

Supplementary Fig. 1. Representative electron density. Stereoview of final refined electron density map for p120RasGAP SH3 domain (purple) in complex with DLC1 RhoGAP domain (teal). Representative region shown in stereoview. $2F_{obs}$ - F_{calc} electron density map contoured at 1σ (blue). F_{obs} - F_{calc} electron density map contoured at +3σ (green) and -3σ (red).

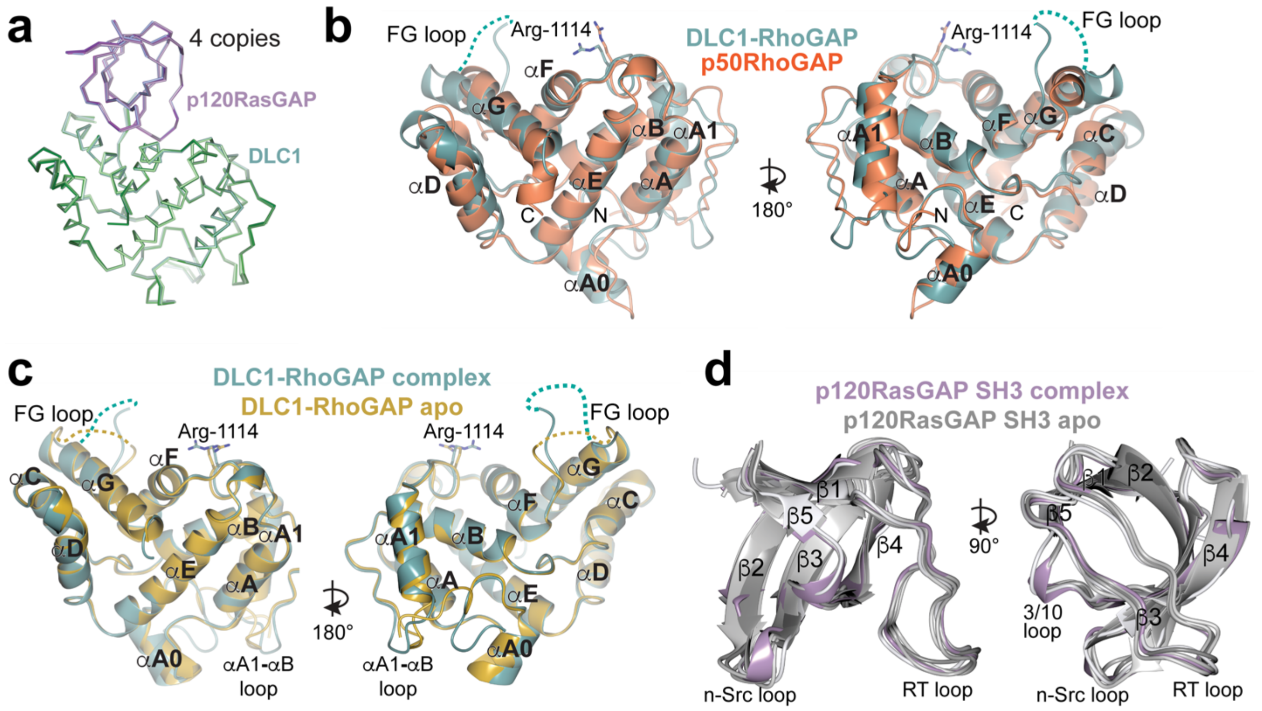

Supplementary Fig. 2. Superpositions of the co-crystal structure of DLC1 and p120RasGAP. **a)** Superpositions of the 4 complex copies from the asymmetric unit. Superposed upon the RhoGAP domains. **b)** Superposition of the RhoGAP domain of DLC1 (teal), superposed with p50RhoGAP (orange, PDB ID: 1TX4 [40]). **c)** Superposition of the RhoGAP domain of DLC1 (teal), with the previously determined apo structure of DLC1 RhoGAP (gold, PDB ID: 3KUQ [39]). The disordered FG loops in (b) and (c) are indicated as a dashed line **d)** Superposition of p120RasGAP SH3 domain determined here (purple) with the previously determined apo structures (various shades of grey) (PDB IDs: 2J05 and 2J06 [43], 4FSS (crystal structure, unpublished), 2GQI (single NMR model shown, unpublished), and 2M51 (single NMR model shown, unpublished)).

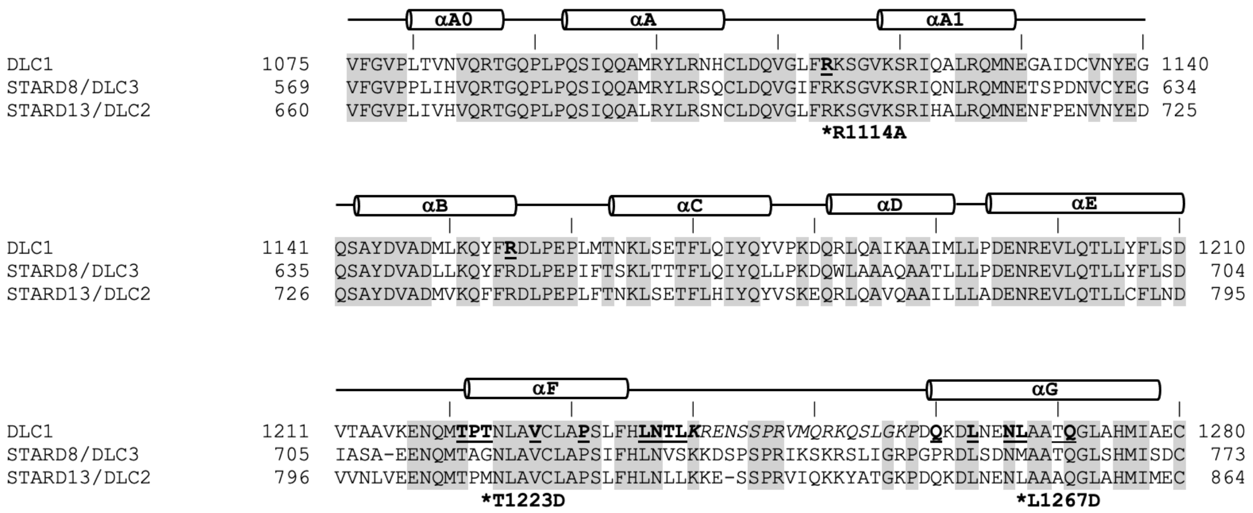

Supplementary Fig. 3. Sequence alignment of DLC proteins. Uniprot ID DLC1: Q96QB1, STARD13/DLC2: Q9Y3M8, STARD8/DLC3: Q92502. Alignment performed in PROMALS [87]. Residues shaded grey are conserved, bold and underlined residues are at the p120RasGAP interface, and italicized residues are disordered in the cocrystal structure.

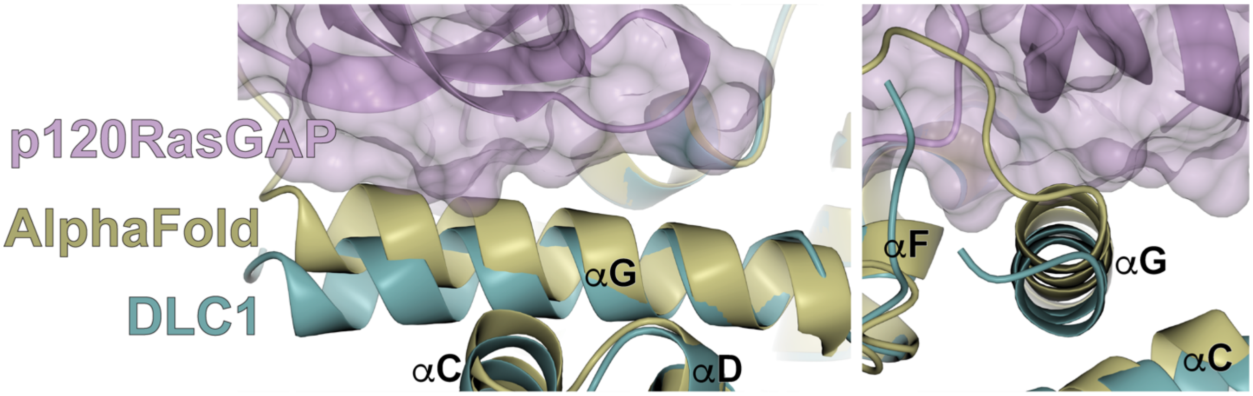

Supplementary Fig. 4. Comparison of experimental versus deep learning model of DLC1 RhoGAP. Superposition of the crystal structure of DLC1 from our structure (teal) with AlphaFold predicted model (khaki, AF-Q96QB1-F1-model_v2.pdb) by superposition on residues 1075-1259, omitting helix αG (residues 1260-1280), resultant RMSD of 0.8 Å over 162 Cα positions (Superpose). The surface of p120RasGAP (purple) is included to show the steric clash with αG in the AlphaFold predicted model.
